# Cell-free mitochondrial DNA and microRNA-137 for early diagnosis of preeclampsia

**DOI:** 10.3389/fcvm.2026.1778221

**Published:** 2026-04-01

**Authors:** Stefanie Marek-Iannucci, Marco Tigano, Caitlyn Cardetti, Shey-Shing Sheu, Maria Gamero Huaman, Rene J. Alvarez, Eduardo Rame, Rupsa C. Boelig, Indranee N. Rajapreyar

**Affiliations:** 1 Advanced Heart Failure and Transplantation, Thomas Jefferson University, Philadelphia, PA, United States; 2 Department of Pathology & Genomic Medicine, Thomas Jefferson University, Philadelphia, PA, United States; 3 Center for Translational Medicine, Thomas Jefferson University, Philadelphia, PA, United States; 4 Department of Maternal Fetal Medicine, Thomas Jefferson University, Philadelphia, PA, United States; 5 Division of Cardiology, Tufts Medical Center, Boston, MA, United States

**Keywords:** biomarker, hypertension, mitochondria DNA, preeclampsia, prevention

## Abstract

**Background:**

Biomarkers for predicting preeclampsia are not routinely available. Currently, preeclampsia is diagnosed with the development of clinical manifestations and proteinuria when widespread endothelial dysfunction and end-organ dysfunction have occurred.

**Objectives:**

This study aimed to (1) compare the serial changes in circulating mitochondrial cell-free DNA (ccf-mtDNA) levels between pregnant women who developed preeclampsia and healthy pregnant controls and (2) to determine if circulating microRNAs (miRNAs) previously linked to placenta development and/or preeclampsia could be detected.

**Methods:**

This single-center, nested case–control study involved singleton pregnancies less than 16 weeks of gestation at increased risk for preeclampsia, with at least one high-risk factor or two moderate-risk factors, and healthy pregnant controls. Patients were followed throughout their pregnancy, with blood collected at three time points to quantify plasma levels of ccf-mtDNA and five microRNAs (miRNAs). All patients received 81 mg aspirin daily initiated before 16 weeks of gestation.

**Results:**

The mean and standard deviation (SD) of age in years was 31.1 ± 7.5 for the preeclampsia group and 31.9 ± 5.7 for the control group. Gestational age at delivery in weeks was 36.3 ± 3.2 for the preeclampsia group and 36.9 ± 4.3 for the healthy control group. The gestational age at diagnosis of preeclampsia was 34.8 ± 3.4 weeks. Participants who developed preeclampsia had a significantly reduced amount of ccf-mtDNA in the maternal circulation compared to that in healthy pregnant controls. The reduction in ccf-mtDNA reached significance at Visit 1 (1.688 ± 1.254 copies for the preeclampsia group versus 4.014 ± 2.381 copies for 1 µL of plasma for the healthy control group), corresponding to the second trimester. ccf-mtDNA levels in the exosomal fraction remained unchanged throughout pregnancy. The plasma levels of miRNA-137 were significantly decreased in patients with preeclampsia compared to healthy pregnant controls at all time points.

**Conclusions:**

Plasma levels of miRNA-137 and ccf-mtDNA are decreased as early as the first and second trimester, respectively before the clinical diagnosis of preeclampsia in the third trimester. Early detection of changes in plasma levels of miRNAs and ccf-mtDNA could be used for early prediction of preeclampsia.

## Introduction

Preeclampsia (PE) is a serious hypertensive disorder of pregnancy affecting up to 8% of pregnant women (1). Its impact on perinatal morbidity and mortality remains high (1). Preeclampsia is mainly diagnosed in the last trimester of pregnancy, with the most common clinical manifestations being hypertension and proteinuria, although its pathogenesis is thought to begin as early as the first trimester (2). Current literature suggests that impaired placentation and an imbalance in angiogenic factors are linked to the development of preeclampsia (2, 3). Furthermore, increased oxidative stress, and therefore, accumulation of reactive oxidative species (ROS), has been associated with the development of preeclampsia by causing impaired placental perfusion leading to systemic inflammation (4). ROS are byproducts of mitochondrial activity in response to cellular stress (5). Circulating cell-free mitochondrial DNA (ccf-mtDNA) has been studied in cardiovascular diseases (i.e., hypertensive disorders) and has been used as a surrogate marker for cellular stress and mitochondrial dysfunction (6). Mitochondrial dysfunction has been linked to the development of preeclampsia, and altered levels of ccf-mtDNA have been described previously (7, 8). In preeclampsia, the data on serial changes in the levels of ccf-mtDNA, a marker of mitochondrial function in preeclampsia, are limited and contradictory.

MicroRNAs (miRNAs) are non-coding RNAs that control gene expression by binding to messenger RNAs (mRNAs). miRNAs can localize in mitochondria and regulate mitochondrial function (9) and play a role in cell proliferation, survival, and metabolism (10). miRNA deregulation has also been implicated in cardiovascular disease, preeclampsia, and cancer (10). Transcriptomic studies have found that cell-free miRNAs may also play a role in the pathogenesis and diagnosis of preeclampsia (11). After a thorough literature review, we selected five miRNAs previously associated with PE, focusing on those associated with altered trophoblast invasion (proliferation or migration), a phenomenon well described in the development of PE (1).

The study aims to investigate potential changes in ccf-mtDNA and miRNAs previously implicated in placenta biology and disease and their potential role in early prediction of preeclampsia.

## Methods

### Patient cohort

This single-center, nested case–control study included 36 pregnant participants and represents a secondary analysis of a subset of participants enrolled in a prospective study. Inclusion criteria for the prospective study were singleton gestations at increased risk for preeclampsia based on at least one high-risk factor or two moderate-risk factors, as determined by the US Preventive Services Task Force (12). Preeclampsia was diagnosed based on the definition proposed by the American College of Obstetrics and Gynecology. It was defined as systolic blood pressure >140 mmHg and diastolic blood pressure >90 mmHg on two occasions more than 4 h apart or systolic blood pressure >160 mmHg and a diastolic blood pressure >110 mmHg and the presence of proteinuria after 20 weeks of gestation. In the absence of proteinuria, preeclampsia was defined by the presence of thrombocytopenia, renal insufficiency, liver dysfunction, pulmonary edema, or headache with visual disturbances in addition to the blood pressure criteria (13).

Cases were participants diagnosed with preeclampsia throughout pregnancy, and controls were those with an uncomplicated term delivery. Participants attended three study visits during which plasma was collected and stored: baseline visit (blood was drawn prior to the first dose of aspirin, <16 weeks of gestation), Visit 1 (18–20 weeks of gestation), and Visit 2 (28–32 weeks of gestation). All participants received 81 mg of aspirin daily. A total of 12 participants with preeclampsia were matched in a 2:1 fashion to 24 controls with a healthy pregnancy.

Pregnant women were followed throughout pregnancy, and blood was drawn at three time points (1): first trimester (baseline) (2), second trimester (Visit 1) (3), and end of second or beginning of third trimester (Visit 2). Plasma was separated from whole blood by differential centrifugation, and samples were stored at −80 °C for further processing. Patient characteristics are displayed in Table 1.

**Table 1:** Demographics of the patient population.

Variable		PE	CTRL	*p*-value
Parity		1.29 ± 1.14	2 ± 2.66	0.307
Gravidity		2.59 ± 1.56	3.48 ± 2.26	0.154
Sex of offspring				0.12
	M	50%	72.70%	
	F	50%	27.30%	
Race				
	White	29.40%	30.30%	0.95
	Black	58.80%	57.60%	
	Hispanic	5.90%	9.10%	
	Other	5.90%	3%	
Age at delivery (years)		31.11 ± 7.41	31.92 ± 5.64	0.84
BMI		34.12 ± 9.06	39.26 ± 9.13	0.067
Smoking				1
	Yes	7.10%	7%	
	No	92.90%	93%	
History of HTN				0.083
	Yes	41.20%	69.70%	
	No	58.80%	30.30%	
History of PE				0.75
	Yes	29.40%	27.30%	
	No	70.60%	72.70%	
Mode of conception				
	Natural	100%	NA	
	Assisted	0%	NA	
GA at delivery		39.19 ± 3.20	36.89 ± 4.29	0.452
PE				
	Early	40%	NA	
	Late	60%	NA	
Birth weight (kg)		2.65 ± 0.57	2.92 ± 0.68	0.596

PE, preeclampsia; HTN, hypertension; (BMI), body mass index; GA, gestational age; CTRL, control. Statistical analysis was performed with a two-way *t*-test for absolute numbers and chi-square or Fisher’s exact test (as appropriate) in case of percentages.

### Circulating cell-free DNA (ccf-mtDNA) and exosomal DNA analysis

**Figure 1:**
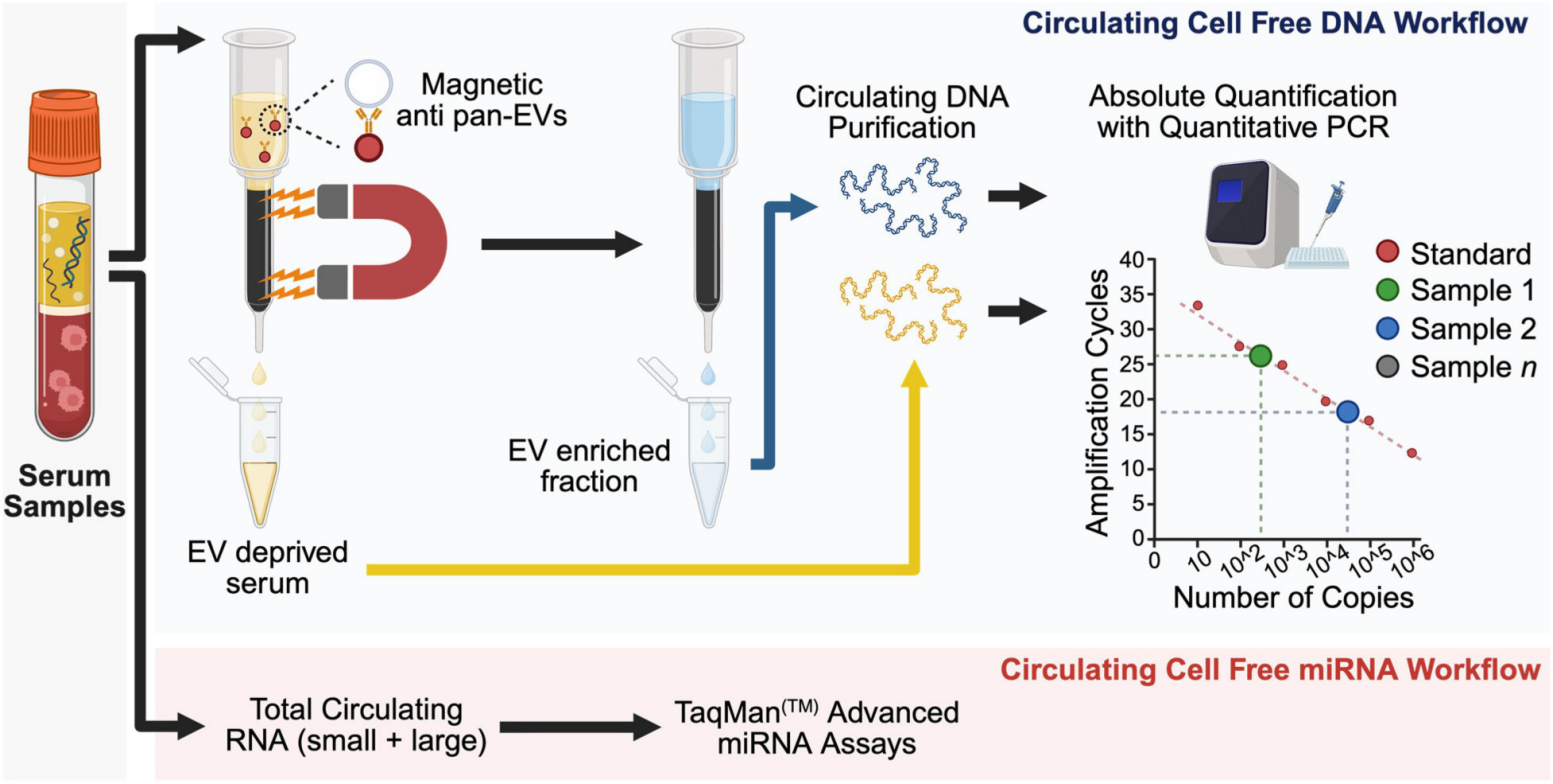
Graphical illustration of sample preparation. Created in BioRender. Tigano, M. (2026) https://BioRender.com/p6d5ynz.

Under normal conditions intracellular mtDNA is found within the mitochondrial matrix, packaged into so-called nucleoids. mtDNA can transit to the cytosol and can be quantified as a marker of mitochondrial stress (14). When mtDNA exits the cell, it is considered an extracellular, circulating damage-associated molecular pattern (DAMP) (15). Extracellular mtDNA can be either cell-free in plasma/serum (also referred to as “naked”) or within extracellular vesicles (EVs) which can be microvesicles (100–1,000 nm) or exosomes (30–150 nm) depending on their size (16).

ccf-mtDNA was extracted from 360 µL of plasma using the commercial kit NucleoSpin cf-DNA XS Micro (Macherey-Nagel) following the manufacturer's instructions. Before ccf-mtDNA purification, plasma samples were diluted with the same volume of phosphate-buffered saline and processed with the EV Isolation Kit Pan (Miltenyi Biotec) to obtain an enriched fraction with extracellular vesicles (EV) containing exosomes. Plasma deprived of EVs was used to purify ccf-mtDNA. EV fractions were also used to evaluate ccf-mtDNA, so that amounts of both naked and membrane-enclosed mtDNA could be evaluated. ccf-mtDNA was eluted in 20 µL, and 2 µL of this was used as template for real-time quantitative PCR (rt-qPCR). For EV fractions, 2.5 µL of each sample was used and boiled for 5 min before amplification to release contents. Mitochondrial D-loop sequence was amplified with specific primers (Fw: 5′-CCCACACGTTCCCCTTAAATAA-3′, Rv: 5′-CGTGAGTGGTTAATAGGGTGATAGAC-3′, Integrated DNA Technologies). TaqMan chemistry (D-loop probe sequence: 5′-ACATCACGATGGATCAC-3′, Thermo Fisher Scientific) was used to perform absolute quantification against a standard curve built to cover from 10 copies to 10e8 copies per reaction vessel. All reactions were performed in technical triplicate. Triplicates with late amplification (Ct > 32) or standard deviation higher than 0.5 Ct were flagged for manual inspection. TaqMan Fast Advanced Master Mix (Applied Biosystems) was used as reaction chemistry on a QuantStudio 5 platform (Applied Biosystems). Analysis and inspection of data was performed with Thermo Fisher Scientific Design and Analysis software (Figure 1).

### Circulating microRNA (miRNA) analysis

miRNAs were selected after extensive literature research and based on previously published data discussing their association with preeclampsia. Circulating miRNAs were extracted from 350 µL of plasma using the commercial kit NucleoSpin miRNA Plasma Mini (Macherey-Nagel) as per the manufacturer's instructions and eluted in 30 µL of nuclease-free water. miRNAs were retro-transcribed and pre-amplified with TaqMan, advanced miRNA cDNA Synthesis Kit (Applied Biosystems) following the manufacturer's instructions (2 µL of input RNA, 14 cycles of PCR preamplification). TaqMan Fast Advanced Master Mix (Applied Biosystems) was used as amplification mix and reactions were run on QuantStudio5 platform (Applied Biosystems). Specific miRNAs were detected with the commercial assays (Thermo Fisher Scientific) listed in Table 2.

**Table 2:** Advanced miRNA assays used in the study (Thermo Fisher Scientific).

miRNA (Reference)	Assay ID/product code	Function
miR-27b (43)	478270_mir/hsa-miR-27b-3p	miR-27b-3p promotes proliferation, migration, and tube formation of human umbilical vein endothelial cells (HUVECs) and enhances invasion of trophoblast cells via regulation of ATP2B1
miR-27a (39)	478384_mir/hsa-miR-27a-3p	miR-27a inhibits trophoblast cell migration and invasion by targeting SMAD2
miR-181a (44)	479405_mir/hsa-miR-181a-3p	Hsa-miR-181a-5p expression was upregulated in preeclamptic placentas and may trigger antiproliferation and inhibition of cell cycle progression, induce apoptosis, and suppress invasion in HTR-8/SVneo and JAR cells
miR-218 (45)	478774_mir/hsa-miR-218-1-3p	Promoting endovascular trophoblast differentiation and spiral artery remodeling and inhibiting trophoblast invasion by targeting LASP1
miR-137 (46)	477904_mir/hsa-miR-137-3p	Proliferation and migration of placenta trophoblast cells in preeclampsia by targeting estrogen-related receptor alpha (ERRα)

### Statistical analysis

After collection, outliers were flagged and removed from the dataset following Tukey's rule (box plot method) in SPSS (IBM). The box plot follows Tukey's rule to identify outliers and extreme outliers using descriptive statistics, including the mean with a 95% confidence interval, quartiles, and the interquartile range (IQR). Outliers and extreme outliers are defined as follows: outliers <Q1 − (1.5 * IQR) or >Q3 + (1.5 * IQR), extreme outliers <Q1 − (3 * IQR) or >Q3 + (3 * IQR). Datasets free of outliers (Supplementary Figure 1) were imported into GraphPad Prism (Dotmatics) to be further transformed and graphed. Statistical significance was calculated with the Kruskal–Wallis ANOVA test with only selected predesigned pairwise comparisons with no further correction. Significance was reached at an alpha of 0.05.

## Results

The mean and standard deviation (SD) of age in years was 31.1 ± 7.5 for the preeclampsia group and 31.9 ± 5.7 for the control group. Two-thirds of the cases had a body mass index (BMI) > 30 kg/m^2^, 25% had a history of diabetes mellitus, and 33% had gestational hypertension. The gestational age at delivery in weeks was 36.3 ± 3.2 for the preeclampsia group and 36.9 ± 4.3 for the healthy control group. The gestational age at diagnosis of preeclampsia was 34.8 ± 3.4 weeks.

**Figure 2:**
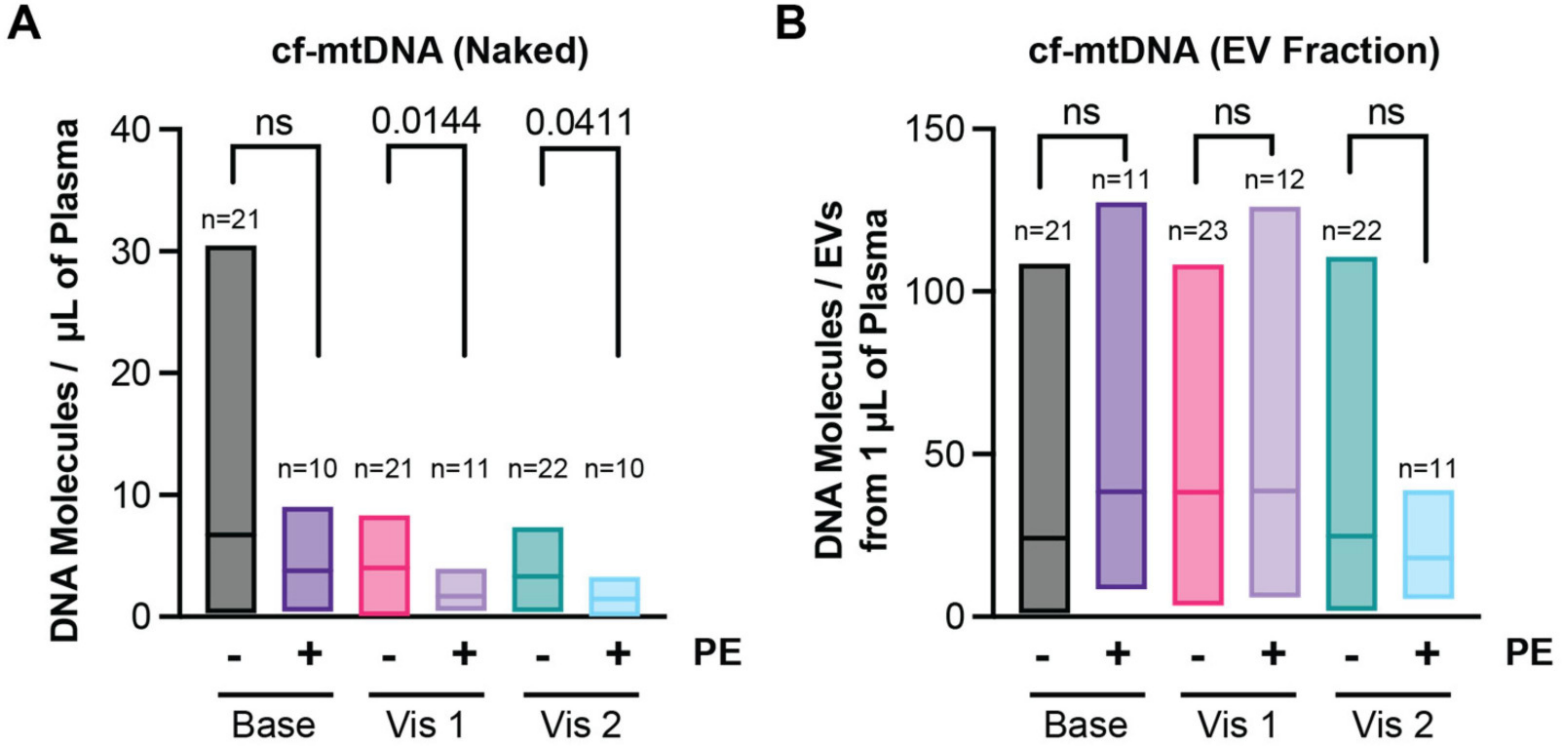
**(A)** Levels of cell-free mitochondrial DNA during the first (Base), second (Visit 1), and third (Visit 2) trimesters in patients with preeclampsia (PE) compared to healthy control pregnancies (Healthy). **(B)** Levels of circulating mitochondrial DNA in the EV fraction during the first (Base), second (Visit 1), and third (Visit 2) trimesters in patients with preeclampsia (PE) compared to healthy control pregnancies (Healthy). Due to the limited number of samples and patient-to-patient variability, data were evaluated for outliers, and statistical significance was assessed using the Kruskal–Wallis ANOVA, without assumptions of normality or lognormality. Only a few predefined comparisons were performed, and therefore no correction for multiple comparisons was applied (also see methods).

Naked ccf-mtDNA was lower throughout the whole pregnancy in patients who developed preeclampsia compared to controls (Figure 2A). The reduction in ccf-mtDNA reached significance at Visit 1 (1.688 ± 1.254 molecules per microliter of plasma in the preeclampsia cohort versus 4.014 ± 2.381 for the healthy control group) corresponding to the second trimester. ccf-mtDNA in the exosomal fraction remained unchanged throughout pregnancy (Figure 2B).

**Figure 3:**
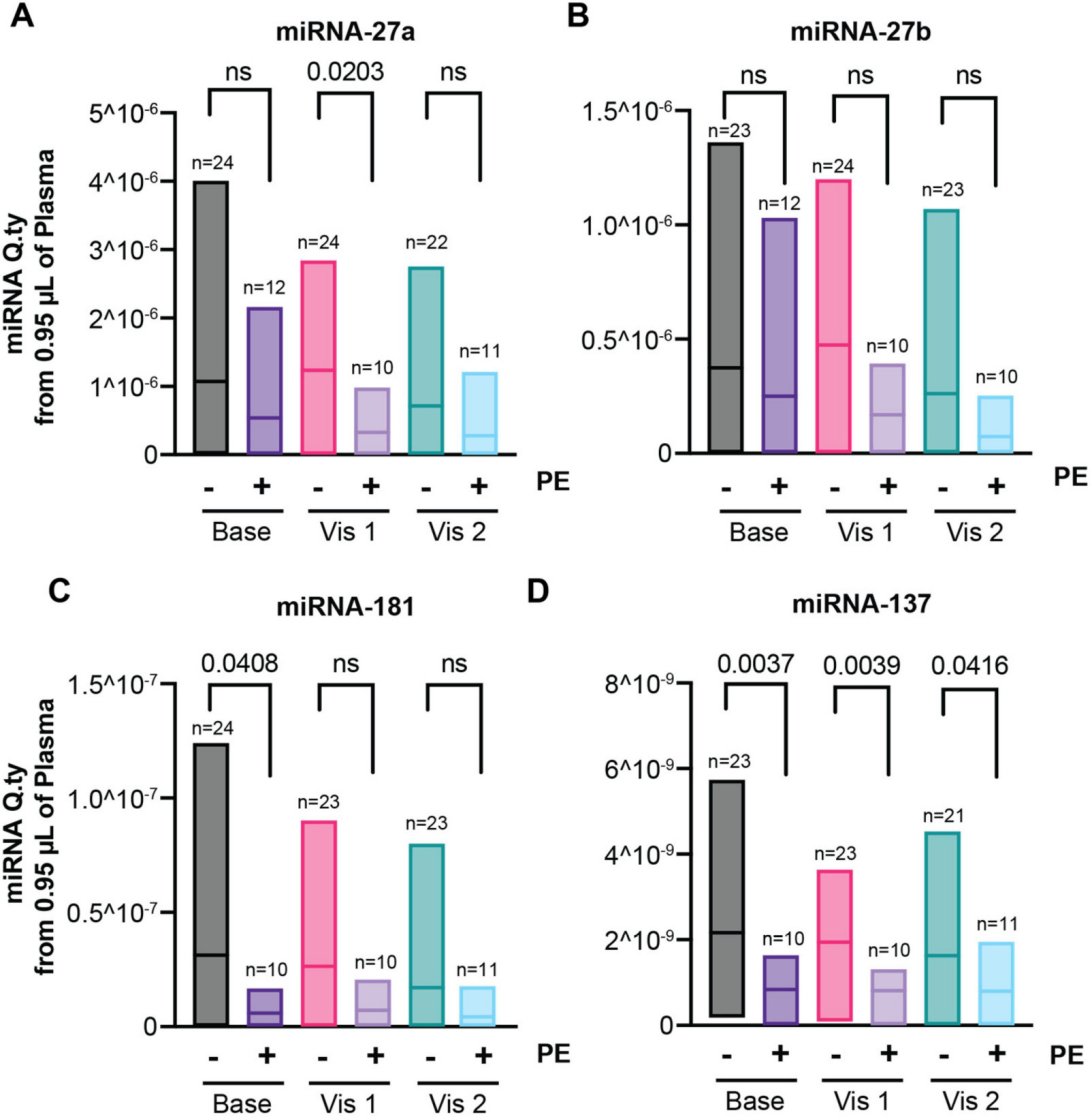
Levels of microRNA (miRNA) 27a **(A)**, 27b **(B)**, 181 **(C)**, and 137 **(D)** during the first (Base), second (Visit 1), and third (Visit 2) trimesters in patients with preeclampsia (PE) compared to healthy control pregnancies (healthy). Due to the limited number of samples and patient-to-patient variability, data were evaluated for outliers, and statistical significance was assessed using the Kruskal–Wallis ANOVA, without assumptions of normality or lognormality. Only a few predefined comparisons were performed; therefore, no correction for multiple comparisons was applied (see methods).

Total and small RNA species circulating were purified from the same samples and used to quantify the amounts of five different miRNAs (miRNA-27a, miRNA-27b, miRNA-137, miRNA-181, miRNA-281) previously implicated in the biology of placenta and trophoblast cells and potentially implicated in preeclampsia (Table 2). While miRNA-281 was undetectable in all samples, a trend toward decreased miRNA-27a, miRNA-27b, and miRNA-181 levels in patients who developed preeclampsia was detected at all time points, but differences were largely non-significant, except for miRNA-27a at Visit 1 (3.286E-7 ± 2.985E-7 in the preeclampsia group versus 1.234E-6 ± 8.976E-7 in the control group) and miRNA-181 at baseline (5.9356E-9 ± 6.3183E-9 in the preeclampsia group versus 3.1318E-8 ± 3.9148E-8 in the control group) (Figures 3A–C). On the other hand, the plasma levels of miRNA-137 were significantly decreased in patients with preeclampsia compared to healthy pregnant controls at all three time points (baseline: 8.3745E-10 ± 5.3798E-10 in the preeclampsia group versus 2.1628E-9 ± 1.4096E-9 in the control group; V1: 8.1322E-10 ± 4.0065E-10 in the preeclampsia group versus 1.9455E-9 ± 1.0165E-9 in the control group; V2: 7.9478E-10 ± 5.6909E-10 in the preeclampsia versus 1.6302E-9 ± 1.2184E-9 in the control group) (Figure 3D).

## Discussion

There is no consistency in literature regarding the type of sample (plasma, serum or placental tissue) or type of measurement (absolute values versus DNA copy number) to quantify mtDNA in pregnant women with preeclampsia. While previous studies using serum samples have shown either elevated or decreased levels of ccf-mtDNA throughout the course of a normal pregnancy and preeclampsia (6, 17, 18), other studies using plasma samples have also described either an increase or decrease of ccf-mtDNA in the circulation of pregnant women diagnosed with preeclampsia (8, 19). Furthermore, previous studies have described an increase in mtDNA copy numbers in placental tissue in preeclampsia (20). In our study, we analyzed the amount of mtDNA circulating as naked molecules (in plasma) or contained within fractions enriched with EVs, well-characterized secretory vesicles with relevance in several disease areas (21). The results of our study showed that patients who develop preeclampsia have a significantly reduced amount of ccf-mtDNA throughout gestation compared to those with a term delivery not complicated by preeclampsia. In particular, the decrease of naked ccf-mtDNA was already detectable and reached statistical significance in the second trimester (Figure 2A). However, the levels of mtDNA in the EV-enriched fraction remained unchanged in pregnant women who developed preeclampsia compared to healthy pregnant controls (Figure 2B). Bradshaw et al. (6) previously published a review regarding the role of circulating mtDNA in inflammatory processes and disease development. The significant decrease in naked ccf-mtDNA in patients with preeclampsia in our study could support this previously published concept and might reflect an attempt to dampen the ongoing inflammatory response during PE possibly through increased uptake of circulating mtDNA into EVs. In fact, previously published data show an increase in mtDNA-containing exosomes in patients with cardiac disease (22). The latter could potentially explain why the levels of mtDNA in the EV-enriched fraction in our study did not follow the same trend as the ccf-mtDNA in patients with PE until the third trimester. The differences in ccf-mtDNA found in our study reached statistical significance in the second and third trimesters as seen in a study by Williamson et al. (19) that showed differences in ccf-mtDNA (in plasma) after 20 weeks of gestation although the levels of ccf-mtDNA in the preeclamptic patients were higher than the controls. This discrepancy in the results could be due to a number of factors, including absolute versus relative quantification strategies or reporting the delta between different time points, as done by Williamson et al. (19). Consistent with our findings, Cushen et al. (8) also showed a reduction of ccf-mtDNA in plasma of women with preeclampsia. Interestingly, they also found higher levels of circulating nuclear DNA and DNase I in women with preeclampsia and concluded that women with PE seem to have altered circulating DNA dynamics (8). This highlights the use of naked ccf-mtDNA as a possible marker for early prediction of preeclampsia in high-risk patients after the first trimester. While these findings are still preliminary, this study serves as the initial step for future studies, with larger study samples, to validate the use of ccf-mtDNA as a biomarker.

Obesity (BMI > 30 kg/m^2^) increases inflammatory activity which in turn affects mitochondrial function and content in placental tissue (23, 24). Recommendations state that BMI alone is not ideal for the diagnosis of obesity, and other components such as muscle strength, performance, and body composition should be considered (25). For the sake of this study, consisting of young women in reproductive age, we considered a BMI > 30 kg/m^2^ as sufficient to classify our patients as obese. BMI was assessed at the first visit. It is important to underline that pregnancy itself has been associated with elevated pro-inflammatory pathways especially in the first and third trimesters (26). In our study, 66% of patients diagnosed with preeclampsia had a BMI > 30 kg/m^2^. Furthermore, 25% of the patients within this study had diabetes mellitus, and 33% had gestational hypertension, both factors associated with increased inflammation (27, 28). Interestingly, mitochondrial DNA has been proposed to be a key immunogen capable of triggering inflammatory responses. While uncontrolled inflammation is detrimental, an intriguing—yet speculative—possibility is that ccf-mtDNA is released in the early stages of gestation to act as a primer molecule, activating compensatory responses that are beneficial during pregnancy. Such a beneficial role for otherwise detrimental molecules has been proposed for mitochondrial ROS, in which a brief, regulated pulse of oxidative stress is required to build cellular resilience by boosting antioxidant defenses, a process known as mitohormesis (29). Similarly, inflammation triggered by ccf-mtDNA could boost organismal fitness during gestation. On the other hand, ccf-mtDNA can act as DAMP, as mentioned above (15), by activating several inflammatory pathways such as TLR-9, NLRP3, and cGAS-STING (30). MicroRNA molecules (miRNAs) can regulate mitochondrial biogenesis and therefore influence mtDNA content and function; on the other hand, stress-induced mtDNA release influences inflammatory signaling, which in turn alters miRNA expression and function in immune/stress responses (31, 32).

Given the above, we further explored the significance of miRNAs in the pathogenesis of preeclampsia. The miRNAs identified for the study were based on an extensive literature search and their connection to PE and mitochondrial dysfunction (Table 2). miRNAs are strongly conserved, small non-coding RNA molecules, which play an important role in gene expression regulation (33). We quantified the amount of five miRNAs (miRNA-27a, miRNA-27b, miRNA-137, miRNA-181, and miRNA-281) in our patient cohort. The latter were chosen as they have been associated with trophoblast migration and dysfunction (Table 2). Furthermore, the miRNAs we chose for the purpose of this study have been associated with mitochondrial turnover and therefore mtDNA content (34). miRNA-27a and miRNA-27b have been associated with the regulation of mitophagy by targeting PINK1 (35). Similarly, miRNA-137 has been associated with the regulation of mitophagy by targeting FUNDC1 and NIX (36). miRNA-181 has been shown to regulate PARKIN-mediated mitophagy as well as mitochondrial biogenesis (37, 38).

While miRNA-281 was completely undetected (data not shown), other miRNAs tested gave reliable amplification. Although a previous study published by Zheng et al. (39) reported elevated levels of miRNA-27a in placenta and serum samples from women diagnosed with preeclampsia at time of delivery, we were not able to reproduce this data within our cohort, as our study did not show a significant increase in plasma samples. This difference could be related to source differences, which may influence expression profiles and interpretation. One miRNA—miRNA-137—displayed a significant decrease in circulation throughout the entire pregnancy and as early as the baseline visit conducted in the first trimester (Figure 3D). Based on this observation, we propose further exploring miRNA-137 to identify patients at risk of preeclampsia as early as the first trimester. Studies have shown that specific miRNAs, such as miRNA-137, are associated with reduced inflammation and oxidative stress in neuronal tissue (40). Reduced miRNA-137 in patients with preeclampsia could play an important role in disease pathogenesis, by promoting inflammation and therefore potential tissue damage, possibly leading to the development of preeclampsia. In fact, previous publications have demonstrated that preeclampsia is associated with impaired vascular remodeling and placental hypoxia, with an increase in oxidative stress within the placental tissue (41).

In conclusion, quantification of ccf-mtDNA and miRNA-137 from plasma could serve as a biomarker for predicting preeclampsia in high-risk patients and the development of targeted therapeutic strategies, which would be a completely novel approach for the management of preeclampsia. It is noteworthy that there is an inconsistency regarding the source of quantification of mtDNA in previous studies as serum and plasma have been used in equal amounts in the past. It might be of interest to directly compare the quantification of mtDNA in plasma and serum of patients to rule out confounding. In fact, previous cancer-related studies did mention a possible contamination of larger DNA fragments in serum compared to plasma (42).

### Limitations

Our study has several limitations. As we are quantifying ccf-mtDNA and the mtDNA content in the EV-enriched fraction, we cannot determine where the mtDNA originates from. This study does not explore the pathophysiological mechanism behind the decreased levels of ccf-mtDNA or the role of circulating plasma levels of miRNAs in placental health and decreasing oxidative stress. The lack of placental tissue to assess mitochondrial content and function is a major limitation of this study. It is clear that the field would benefit from agreement regarding the best strategies to quantify and report the data.

## Data Availability

The authors acknowledge that the data presented in this study must be deposited and made publicly available in an acceptable repository, prior to publication. Frontiers cannot accept a manuscript that does not adhere to our open data policies.

## Supplementary material

Supplementary Figure 1 Tukey's removal of outliers and extreme outliers. For each analyzed molecule and time point, descriptive statistics were generated in SPSS (IBM) to present the mean with 95% CI and to calculate the quartile (Q) distribution and interquartile range (IQR). Tukey's removal (boxplot method) defines outliers (round circles) as values <Q1 − (1.5 * IQR) or >Q3 + (1.5 * IQR) and extreme outliers (stars) as those with <Q1 – (3 * IQR) or >Q3 + (3 * IQR).
