# Supplementary figures and images for: Cell-free mitochondrial DNA and microRNA-137 for early diagnosis of preeclampsia
Source: Front Cardiovasc Med. 2026 Apr 1;13:1778221. doi: 10.3389/fcvm.2026.1778221 (PMC13078980; doi:10.3389/fcvm.2026.1778221)

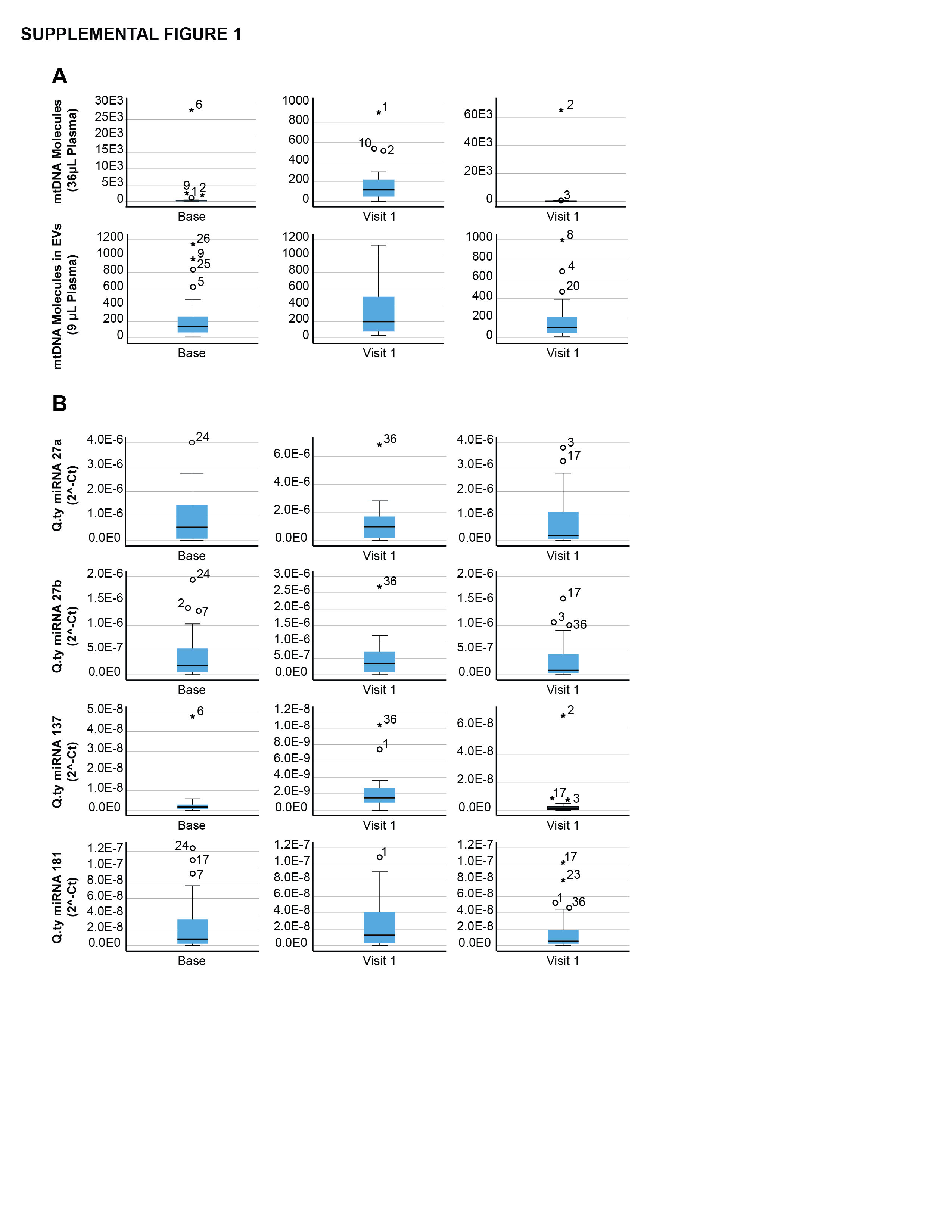

Supplement: Supplementary Figure 1 — Tukey's removal of outliers and extreme outliers. For each analyzed molecule and time point, descriptive statistics were generated in SPSS (IBM) to present the mean with 95% CI and to calculate the quartile (Q) distribution and interquartile range (IQR). Tukey's removal (boxplot method) defines outliers (round circles) as values Q3 + (1.5 * IQR) and extreme outliers (stars) as those with Q3 + (3 * IQR). [file Image1.png]
